# Information Needs of Australian Families Living with Craniosynostosis: A Qualitative Study

**DOI:** 10.1177/10556656241298813

**Published:** 2024-11-21

**Authors:** Amanda J. Osborn, Rachel M. Roberts, Diana S. Dorstyn

**Affiliations:** 1 School of Psychology, 1066 University of Adelaide, Adelaide, SA, Australia

**Keywords:** craniosynostosis, craniofacial, information needs, support

## Abstract

**Objective:**

Craniosynostosis is considered a lifelong condition, yet relatively little focus has been directed toward ascertaining the information needs of Australian families. Thus, the aim of this study was to explore the information needs of Australian parents whose child has been diagnosed with nonsyndromic or syndromic craniosynostosis.

**Design:**

Twenty-one online narrative interviews were conducted with parents of children with craniosynostosis (aged between 4 months and 20 years). Transcripts were analyzed using reflexive thematic analysis and themes were developed.

**Results:**

Four themes were generated: (1) lots of information …. and quickly!; (2) the practicalities of hospital and surgery; (3) guidance on how to talk about my child's condition; and (4) the path is rarely clear. Parents of children with craniosynostosis discussed a range of information that was provided to them, or they would have liked to have been given, following their child's diagnosis. Parents noted that insufficient information was provided by the health system and that they faced considerable difficulties accessing credible information about their child's condition, relevant location-specific surgical options, the treatment process and outcomes.

**Conclusions:**

Narrative interviews provided detailed insight into the information needs of Australian parents of children diagnosed with craniosynostosis. Although parents were frequently challenged by a lack of information detailing their specific treatment and support options, suggestions relevant to craniofacial providers globally were offered. Further work is now needed to develop and provide these information resources in a timely and easily accessible way.

## Introduction

Craniosynostosis is a typically congenital (ie, present at birth) condition that occurs when one or more of the cranial sutures that separate the bony plates in the skull fuse prematurely. Early fusion impedes the normal growth of the skull and usually requires surgical treatment to reduce potential risks to the child's neurodevelopment and prevent the resulting anatomic malformations of the skull.^1^ Approximately 90% percent of craniosynostosis cases are categorized as nonsyndromic and, after comprehensive dysmorphologic and genetic evaluation, the remaining cases are considered syndromic, which are associated with additional abnormalities.^2,3^ Global estimates indicate that nearly 85 000 children were born with craniosynostosis in 2019,^2^ noting varying rates due to differences in maternal exposure to risk factors (eg, malnourishment, hormone imbalances, pollutants), physician awareness of craniosynostosis, and the quality of health-care and treatment available.^2,4–6^

Despite this large number, and the growing evidence of high levels of burden and distress experienced by these parents,^7^ few resources have been developed in order to assist families in accessing quality and reliable health information, or to support them on their craniofacial journey. The American Cleft Palate-Craniofacial Association's (ACPA) Parameters of Care guidelines note that the achievement of treatment goals for a child with craniofacial differences requires periodic assessment of their psychosocial needs, as well as the needs of their family.^8^ Their recommendations include providing parents and children with information on such topics as: anxiety and expectations around surgical treatment; negative reactions from both within and outside the family; and emotional adaptation to treatment. Moreover, providing families with adequate information is an important aspect of patient-centred care because it increases treatment satisfaction, aids decision-making, and improves health-related outcomes - including psychological well-being and quality of life.^9^ Ascertaining the support preferences and needs of parents and children is therefore critical in order to deliver a high-quality patient-centred health service.

Notably, the practice of consulting with specific patient groups about what resources and information would be helpful to them is still not common in Australia.^10^ Parents of children diagnosed with a craniofacial condition have reported that information is difficult to access and not always relevant, as it is sourced from international organizations, with similar information concerns being expressed by parents of children with craniosynsotosis living in the UK.^7^ As such, families often struggle to find credible, evidence-based resources over the weeks, months and years following their child's craniofacial diagnosis. Problems can be exacerbated for those residing in regional or rural areas with limited ongoing support, potentially resulting in adverse psychosocial outcomes for the broader family unit.^10^ Addressing parental information needs is critical given the potential psychological impact of a craniofacial condition on the affected child, their parents and siblings.^11^ Indeed, a key risk factor for poor medical and psychosocial outcomes in children with a chronic illness is parental psychological distress.^12^

To address this research gap, we consulted with Australian parents about what resources, tools and information they would have found useful during their family's craniofacial journey. Using qualitative methodology, we were able to access the valuable experiences of parents of children diagnosed with craniosynostosis and, in turn, generate recommendations for improving the dissemination and quality of information provided to these families. Our primary aim therefore, was to explore the information needs of parents and children diagnosed with craniosynostosis in the context of the Australian health system.

## Method

### Participants and Ethical Considerations

Following institutional ethics approval by the University of Adelaide Human Research Ethics Committee (project approval number: H-2023-162), various communication channels (paper-based, email, social media) of a national foundation that supports the craniofacial community through patient care, education and research, was used to recruit families from across Australia. Parents who expressed an interest were emailed a Participant Information Sheet and Consent Form. All who subsequently provided written informed consent were interviewed. Additionally, verbal consent was obtained at the commencement of each interview. Each family was provided with a $AUD 60 gift certificate as recompense for their time. All participant information was anonymized and pseudonyms were allocated, in order to ensure confidentiality.

Overall, 22 families participated in interviews. Nineteen children had nonsyndromic craniosynostosis (sagittal, metopic, unicoronal, or bicoronal synostosis), 2 had been diagnosed with syndromic craniosynostosis (Crouzon, Muencke) and one with bilateral cleft lip and palate. At the time of the interview, children were aged between 4 months and 20 years (12 males, 9 females, 1 nonbinary individual). Twenty-one interviews were conducted with mothers only, while the remaining interview included both the child's mother and father. The data from the mother whose child was diagnosed with bilateral cleft lip and palate (male, 7 years old) were excluded from this analysis as this condition can require different information needs and support (eg, support with feeding and lactation).^13^ This analysis therefore includes data from 21 families (22 parents) impacted by craniosynostosis, with all children having undergone surgery, except for one child who was in the pre-surgical phase of their treatment.

### Dataset Generation

In order to optimize the depth and quality of information provided by parents, a series of open-ended questions relating to each parent's experience of support during their craniofacial journey and at different life stages for their child were developed by the authors. Specific stages examined were at: diagnosis, surgery, infancy (birth to 23 months), toddler age (2–4 years old), primary school age (5–11 years old), secondary school age (12–17 years old) and adult (>18 years old). Questions were based on recent literature^7^ and the first and second author's extensive research and clinical experience with parents of children with a craniofacial condition, and reviewed by a clinical psychologist (third author) experienced in medical rehabilitation settings. Essentially, the 2 central questions asked were: (1) can you tell me about the information and support you received when your child was (insert stage) that helped you cope with any difficulties you were facing? and (2) what information or support, that you didn’t get, would you have found helpful at (insert same stage)—and how would you have liked it to be provided?

The first author conducted the semi-structured interviews, which began with a brief summary of the study, in addition to highlighting that the interview was being video recorded. Basic demographic and clinical information were then collected, followed by the aforementioned broad questions, with the interviewer clarifying responses as necessary and, at times, prompting for additional information. For example, whether parents received information on topics such as coping or resilience and, if so, asking whether they sought this information themselves or if it was automatically provided, as well as what stage of their craniofacial journey they received the most information and support. Each participant was encouraged to expand on their response in any direction important for them. Interviews were conducted on an informal basis (eg, conversational tone, use of first names, casual attire) in order to address any perceived power imbalances and encourage participants to speak openly and honestly about their experiences. The interviewer was known to some participants as they had participated in earlier craniofacial research, but no conversations or meetings had previously taken place, nor was any member of the research team involved in their clinical care.

Interviews were undertaken between August and November 2023 and ranged between 33 and 99 min, with an average length of 49 min, and totaling 18 h across 21 interviews. One interview was split over 2 consecutive days due to participant availability. Interviews were conducted online in a face-to-face format and were video- and audio-recorded with participant consent. Interviews were arranged for a time that was convenient for parents, with some participating from their homes and others from private settings such as their car or work office. Interviews were recorded via Zoom and an automatic transcript was generated. These auto-generated transcripts contained translation errors, so the interviewer used this opportunity to replay all interviews in full. This enabled the interviewer to further contemplate the issues discussed, in addition to amending words that were incorrect. Transcripts were additionally forwarded to the second author for consideration, with particularly traumatic parental experiences discussed between the first and second authors in order for the interviewer to debrief. On 2 such occasions, where the interviewer had gained permission from the participant, the second author, a clinical psychologist, contacted mothers to advise of avenues of support. Transcripts were also provided to each parent for their information, as per the study's ethics approval. At this time, we offered that parents could amend, clarify or add further information if required, with one parent advising of a single minor typographical correction.

### Data Analysis

In view of the limited understanding available from the previous literature, and in order to collect detailed, rich data to inform service provision, an exploratory, qualitative approach was undertaken. We used reflexive thematic analysis^14–16^ to analyze the data provided by parents and generate themes (ie, patterns of shared meaning) that reflect the experiences of parents in accessing information during their craniofacial journey. To this end, our reflexive thematic analysis qualitative approach involved an experiential and contextualist theoretical perspective whereby we were giving voice to each participant's contextually situated experience, while also acknowledging that the views and experiences expressed were each individual's “reality.” Meanwhile, our analytic approach was inductive (ie, was grounded in the actual data provided by the individual) and semantic because we were looking at the surface level of content provided (ie, the data are observational and descriptive).

Reflexive thematic analysis differs from other forms of thematic analysis in that it emphasizes the importance of the researcher's reflexive engagement with participants, the information provided and its interpretation. In the current study, the researcher who conducted the interviews and analyzed the subsequent data has interacted with many parents of children with craniosynostosis, in addition to undertaking cognitive, behavioral, and psychological assessments of children themselves. Her background is also steeped in long-term use of the medical system, both as patient and carer, and she has experienced life-changing positive outcomes when treatment is successful, in addition to the frustrations and challenges that can arise in medical settings. This experience inherently influenced identified themes.

The approach taken for the current study was based on Braun and Clarke's recent explication of reflexive thematic analysis.^16^ This approach is not linear, but requires an ongoing and recursive process involving becoming familiar with the data, researcher reflexivity, data coding and theme generation (followed by theme review, refinement, defining and naming) and, lastly, report writing. Themes are developed based on central concepts and shared meanings across participants and are formed into coherent and related “outcomes.” In the current study, themes were generated based on parental perception of the required information that should be available to families with a child diagnosed with a craniofacial condition. Themes are additionally represented with interview excerpts from participants.

## Analysis

Four themes were identified from the data: (1) give me lots of information … and quickly! (2) the practicalities of hospital and surgery; (3) guidance on how to talk about my child's condition; (4) the path is rarely clear. These themes reflected the information needs of parents throughout their family's craniofacial journey. Experiences of support across Australia were highly variable, with the psychological well-being of parents often seemingly linked with their perception of the quality and volume of information provided by the medical system. Notably, many parents had never heard of craniosynostosis when their child was diagnosed and struggled to access information about its characteristics and treatment. Families also approached this task differently. Some were guided by clinicians to specific resources, while others were left to source information without assistance. Although the individual journey for each family in the first few months and years after their child's diagnosis was different, a common theme developed around the type of information that would have been helpful and may have made their experience less traumatic.

### Theme 1: Give me Lots of Information … and Quickly!

Parents struggled, albeit in different ways, to learn about their child's condition and its implications, with many noting that their health service had provided limited information. They often drew on their own medical or personal experiences, or relied on informal networks, to obtain adequate information. For example:

Luckily, one of our nurse friends was able to get an information pack…. and she gave me that, and that's where I learnt about the different types of treatment available, but from the health professionals there were 6 weeks where we got absolutely nothing, so that was, it was hard. (P9)

Nevertheless, while parents were generally satisfied with the medical care that their child received, they felt that the surrounding clinical process of communicating appropriate and timely information was often lacking. Not understanding how the process would unfold and generally having little control over events led to increased parental anxiety, with one mother noting that:

it was the medical information that helped us cope. (P6)

Moreover, parents often felt abandoned by the health system at a critical time in their baby's life, particularly in cases where there was a long delay from diagnosis to surgery. The enjoyment of having a newborn baby was negatively affected because, instead of joyful family moments, those early months were often filled with grief around what their child and family was experiencing:

I feel like I missed out on the newborn stage, because the entire time from, you know, 15 weeks to 15 months, I was waiting for that huge operation. We weren't provided sort of, any kind of, information on it, no sort of, you know, no reliable source for us to go and research. (P21)

Moreover, there were feelings of maternal guilt and anxiety because mothers were worried, and/or had been blamed by family members, that perhaps the condition had been caused by their actions during pregnancy.

is it something I've eaten, or something I do in my day to day life that affected it. (P10)

Although parents would find and read information online, they often did not know whether the information was applicable to their child (eg, differences between sagittal and metopic synostosis). Parents were often left to their own devices and the volume of potentially useless information they consumed was immense, leaving them feeling overwhelmed and anxious.

at the diagnosis stage, it would have been nice to be given some information packs on … just an overall look at what it is and what they do, how it's treated. (P9)

Regrettably, even when information was provided, parents would receive vast quantities of technical information verbally, without the added benefit of written information that they could reflect on later. Families also found it difficult to reconcile the conflicting information between what they read online versus what they were subsequently told by surgeons, which only added to their stress.

I read about the research and outcomes, and I found a range like the, the cephalic index is supposed to sit in with for example. And one of the surgeons told me, no, no, we don't use that. Don't worry about it. We don't measure heads. (P5)

Currently, there is little Australian specific information about treatment of different types of craniosynostosis, with parents directed to US-based websites on the advice of health professionals:

essentially, she said to us, look, don't Google anything. I'm going to quickly Google it for you. And here is an article that I suggest you look at, because if you look it up yourself, you're going to see horrible cases out there. You're going to see things that don't apply to his current condition. So have a look through this article. It was just a generic article, I think, like the first or second search that comes up just explaining what the condition is, and it was an American one, too. So surgery is a little bit different over there, so they don't have some of the options that we do, and the timings are different, and yeah, so it was just basically a summary of what the condition was and what they do overseas. And that was it. (P5)

As the pediatrician in the above quote noted, random internet searching often returns results that are highly distressing for parents. These may be the first websites parents visit, possibly not long after their child's birth and the images are indeed often confronting. Particularly when the specifics of their child's condition are likely not yet formalized, and the parents themselves are uncertain which of these photos represents the particular type of craniosynostosis that their baby has. As one mother noted below, there are many “worst case scenarios” on the internet, but when you are already in a fragile state of mind, it is difficult to be a “prudent consumer.”

I think, what are we, we’re so, we want to search, we, we search for knowledge and information because we think that knowledge and information is power, and that actually the, the rational part of me knows that there's a lot of misinformation on the web. That I need to, I need to be a prudent consumer… To have someone filter that for me, would have been useful at the time. If you’re going to Google, here are 3 really credible research-based things that you can use. (P12)

In addition to learning about their child's condition, parents were often required to make decisions around the type of surgery (eg, calvarial vault remodeling [CVR], spring-assisted cranioplasty, strip craniectomy), that their child would undergo. They were often bewildered by these options which, even within specific techniques of the same name, can differ across Australian states (eg, is it better to use absorbable or nonabsorbable surgical materials during a CVR?). Some parents also considered whether traveling overseas for surgical techniques not practiced in Australia was actually the “correct” decision for their child, despite the huge logistical and financial burdens that course of action would pose on their family.

in regards to the different surgery options, there's so much debate about what's better and, you know, so me, I was considering going to Boston. But then, you know, to do the helmeting, it's like possibly a year of them wearing a helmet, and I couldn't move my family to America for a year. I know different other families that fly the helmets back and forth, and I was just like, oh, this is just getting crazy. And money as well, you've got to factor in, it was expensive enough, we had to borrow money from my friends. (P19)

Nonetheless, some parents were not given a choice and the surgeon in their locality dictated the surgical process to be used based on the types of surgery they personally recommended and performed. Surgical decisions were often time-critical, with different types of surgery being performed at different stages of the brain growth curve. So, for parents given choices, they often felt under immense pressure to decide quickly on a particular course of action, knowing that their choice might dictate their child's well-being for the rest of their life.

But what struck, struck me, that was really difficult to cope with, is that they put the choice to you as to which surgery to do. So it's a really heavy burden of, are you making the right decision for my child, because I could choose the wrong thing and the consequences could be, you know, life altering. (P5)

### Theme 2: the Practicalities of Hospital and Surgery

Another common thread voiced by parents concerned the difficulties and uncertainty that revolved around the practical aspects of attending hospital with their infant, being psychologically prepared for changes in their child's appearance and knowing how to care for their child when they were discharged from hospital.

Many spoke of how their need for this type of information was fulfilled by an Australian online Facebook support group created by, and comprised only of, parents. This group's aim is to provide support to other families by offering practical and psychological assistance. This help was spoken of as invaluable, with multiple parents stating that this parental Facebook support group was either their only, or most valuable, source of information.

I would say that the, probably the most helpful information we got came from the Facebook group and it would have been great for some of that to be formally provided to every patient going through. (P8)

Practical assistance provided by the Facebook group included advice about what to bring to the hospital, answering hospital-specific questions such as where food was available, and whether they could use a kitchen or have a shower. Seemingly small concerns such as what clothes to pack for their baby became important, as these practical issues were some of the few elements within parent's control. Although this information may be supplied by hospitals, it is commonly generic advice such as “bring comfortable clothes,” as opposed to custom advice for an infant who has just undergone head surgery, is heavily bandaged and physically cannot have clothes pulled over their head. Others lamented finding out after multiple visits to the hospital that there were financial discounts available on nearby accommodation options and car parks:

and I was like, god, we spent hundreds of dollars on that bloody carpark, like just that information would have been helpful. (P20)

The over-arching perception of parents was that the practical information provided by peers on the Facebook support page was much more detailed and grounded in experience than information available from hospitals.

Parents had mixed experiences in regard to how adequately clinicians had prepared them for changes in their child post-surgery and given them support in how to care for their child post-discharge. Some mothers expressed anxiety, distress and grief around the lack of preparation for the physical changes that would occur and the potential for their child to look different, for example:

I don’t think they prepared us enough for how different he looked after surgery, sagittal, his whole face changed and I think that would have been good if someone had have prepped me for that. Because he was 6 months old after the surgery and he was still feeding at night and it felt like I was picking up an alien, like it didn’t feel like my baby, I had to get to love the new face. (P6)

On discharge from hospital there was parental anxiety about practical issues such as how to monitor wounds and manage sleep problems, and what ongoing symptoms are indicative of issues that would require further medical attention.

there's like a real integration process as a family where all of the routines and rhythms that we’d had at home got totally mucked up. And we were able to re-establish them, but it was a long process of getting back to normal which we didn’t expect. (P8)

The Facebook support groups were again important at this time because other parents were able to offer practical advice such as:

expect one week of disordered sleep for each night her child spent in hospital. (P8)

This informal transfer of relevant and helpful information was particularly invaluable for parents and helped them manage their own stress and anxiety.

Other practical information that parents would like to see provided by clinicians was how best to navigate the public and private medical system in Australia, and the differences between these 2 treatment options. While the Australian medical system includes a basic universal health insurance scheme that provides free or subsidized access to craniofacial surgery, during the COVID pandemic free public elective surgery (using the public system) was canceled in some locations. These families were often frantic, having been told that surgery for their child was time-critical. Some transferred to the private system which involved greater expense, but enabled their child to receive timely treatment. The lack of readily available information about the practical differences between these 2 options (public vs. private treatment) made decisions more difficult.

We have a private health fund. So we opted to go private to help the hospital out, and you don't get much back for it, like you get a parking voucher and, you know, some meal vouchers. But even that, we weren't aware of the fact that that was kind of an option, or what it was all about, didn't understand it until we're actually in that moment doing it. (P10)

### Theme 3: Guidance on how to Talk About my Child's Condition

Another area that parents struggled with and felt that information provision could have been improved, was learning how to discuss their child's condition with others. These difficulties were encountered across a broad variety of interactions including how, and when, to talk about it with their affected child, siblings, family, friends, strangers & daycare centers/schools. Perspectives on how parents approached this issue varied from attempts to provide technical specifics of the condition:

but no-one really understands it, and it is a lot to kind of explain and a big word to say. (P10)

Through to a broader, more existential approach such as:

what do you say when some-one says, what happened? How do you even begin to describe the journey you’ve been on? (P12)

Not only did parents often not have the words to describe what was happening but, although others usually meant well, the inquisitiveness of strangers often re-created feelings of trauma as parents re-lived their craniofacial journey each time someone questioned them. Sometimes however, these encounters were less well-meaning and parents suggested that information could be provided so that they have some resources to draw on in order to:

prepare for and address ‘the starers’ or people questioning when out in public, or when another child at a playground is saying to your child that they look different. (P1)

Some parents endeavored to use avoidance strategies in order to minimize attention. While multiple mums mentioned the benefits of hats to hide scars caused by head surgery, other families chose to segregate themselves and avoid social contact.

I didn't want questions when I went out, so as soon as we could put a hat on, we put a hat on him, and off we went. (P15)

Conversations within the family environment were also noted as being difficult at times. Some parents found it challenging to speak with their child about what had happened to them and either did not know how to articulate their child's journey, or were unsure about what information to convey, and at what age. One mum noted that it would be helpful to receive information and

guidance on how to build this surgery into your child's narrative. (P16)

There was also uncertainty about what might be an appropriate strategy for discussing what had happened with their child. Families approached this task in different ways—often dependent on whether the child's condition was visible as they aged. Some considered their child's surgery to be an event in the past and did not discuss it with their child at all. Others created resources such as picture books incorporating photos of their child, to use as a starting point for discussing what had happened. The over-arching message, however, was that some information about the best way and time to raise this with their child, would be highly valued.

Related to this was the problem of how to explain their child's condition to siblings and support siblings through the process. For a year or 2, the child in the family with craniosynostosis was often the focus, with constant medical appointments, and parents consumed with ensuring that their child was receiving the best treatment possible. Nonetheless, in families where the child had siblings, parents needed a different set of resources in order to smooth the integration process and minimize distress for the sibling.

I wish that I'd had someone else to talk to and draw from, around how do I support this older sibling through this process too? (P7)

Another common issue that was raised by parents of older children, was how they had managed the process of their child starting daycare and/or school. Some families had already been discharged by their craniofacial health care clinicians, while others were still having regular monitoring. Parents expressed their desire to be provided with information and guidance on how to prepare these formal organizations for their child's attendance. They wanted those settings to be prepared appropriately, parents wanted information on how best to:

advocate for their child, in order that they would be supported and respected in new environments. (P1)

Some parents attempted to inform and educate organizations themselves, preparing orientation letters and including fact sheets about craniosynostosis from overseas organizations. They also talked with teachers about how to raise awareness amongst other children and sourced story books from overseas that could be read in class. One mother arranged for their child's surgeon to write a letter at the start of the year outlining what had happened to her child and what staff should look out for. In contrast, other parents felt that once their child had recovered from surgery that this type of intervention was not required:

it's kind of like, once the surgery happened, he'd recovered, yep we've got these regular appointments to check, but it's done and dusted. It's no longer a thing. (P14)

### Theme 4: the Path is Rarely Clear

A lack of information about what was about to happen, at every stage of their craniofacial journey, contributed to the anxiety experienced by parents. When their baby was diagnosed, parents understandably became focused on the specifics of their child's treatment. Families’ lives were often consumed by specialist appointments, learning about the specific condition, and attempting to understand how their child would be affected long term. They constantly sought reassurance that everything would turn out okay in the end, but in the absence of credible and reliable information, it was difficult for them to feel confident about the way forward. Families spoke about their frustrations trying to navigate the process because there was so much that was unknown and often either little information, or verbal information only, had been provided by health professionals. Parents spoke about how having an overview or “roadmap” would have helped prepare them for the process that was going to unfold:

once he was diagnosed, it would have been really nice to just see step number one, step number 2, you know, this is what to expect. I certainly didn't realize how much time we were going to be spending in the hospital. I felt like 2 months of our life was spent in waiting rooms. (P9)

This lack of information and subsequent understanding often led parents to feel they had little input or control. Nonetheless, while parents acknowledged that too much information could be overwhelming, they did want to be actively involved in their child's treatment. They wanted to understand what was happening and the reasons why. For example, one mother said:

Cause, I guess, for me within it, still having a choice was important, as opposed to just being told you're seeing this person. Yeah, without giving me a rationale for it as well. Like, if someone explains why, like so I mean, a lot of, we were like, oh, why do we see the speech pathologist, or this person or that person, but that became clear when we saw them. Oh right, their role is this, this is what they do in the team. (P1)

Compounding this difficulty was the fact that some women were first-time mothers, leading to an even steeper learning curve than they had anticipated. Not only did new parents require support and information about caring for their new baby, but they also had to deal with a diagnosis of a craniosynostosis and all of its ramifications.

So there was so much you were trying to process, let alone with a new baby to organize… She was 6 weeks old, I mean, I don't even know that you've got a handle on breastfeeding or babies at 6 weeks to be fair, you know. (P12)

Parents also felt unclear about surgical outcomes and, again, used social media to access relevant information and communicate with families who had lived through similar experiences. Families would post stories about their children's progress, including photos of immediate post-surgery stages to help “new” families understand what the future held. They also provided social support and reassurance to other families about to embark on surgical treatment.

online, online were very good, and online people, they still put photos like, for people going through it, like this is what they look like… I think the surgeons would have told us, but I felt like online was more, would speak at our level, you know. (P6)

When families posted online photos of how their child progressed over many years following surgery, it gave other families hope and more clarity around the fact that their child would be ok.

I think medically, I understand that we have to analyse risk or talk about worst case scenario… the Facebook group…does this really well. You can see a photo of a child at 3, 6, 12 years, 15 years, because all I saw was internet worst case situations and what it might look like, and what the deformity would be, or what the intellectual disability would be, or, you know, a series of, of worst case scenarios really. (P12)

It's understandably difficult for medical staff to be prescriptive about all the risks that may be present post-surgery, but even when parents were given guidelines, there was still confusion about what constitutes “enough”? How frequent and how severe did headaches need to be before parents rushed their child to the emergency department? What constitutes a head knock serious enough to seek treatment? Should they be treating their child any differently to other children at this stage anyway?

and then just monitoring his development afterwards, because they say, you know, there could be a chance that there's pressure on the brain. So you're gonna have to continue to monitor him, but they've never really said to us, apart from, you know, not meeting milestones. What that looks like when he's older. (P5)

Parents also spoke about not understanding the path forward in terms of ongoing post-surgery management. Moreover, given that surgical units across Australia have differing treatment protocols, these ongoing care plans are likely to differ for patients in different localities.

I don't know what the care plan is, so how often are we going to see these doctors moving forward once the helmet is off? Just more of a roadmap. Yeah, of what the surgery looks like and what the aftermath is gonna look like. (P5)

Another common area of concern for parents was the lack of long-term information available about the effects of craniofacial conditions (ie, esthetic, cognitive, behavioral, psychological). While parents acknowledged that research was scarce, clinicians rarely discussed longer-term functioning, leaving them uncertain about their child's future:

probably not enough information about the psychosocial development of the child either and whether it would impact on the child… are there going to be, yeah, any sort of long-lasting cognitive, behaviour, sort of impacts? (P17)

Mothers were anxious and distressed about this unknown developmental path and worried at how well their child would survive and thrive. Providing more information about this trajectory would greatly assist families.

I just think there needs to be some more information about what does the road look like over the next 3 to 5 years, to give some reassurance that your child can live a normal life, they can be happy and thriving. (P16)

## Discussion

We interviewed 22 parents of children with craniosynostosis from across Australia in order to understand the information they were given, or would have liked to be provided, during their family's craniofacial journey. The quality and volume of timely and appropriate information provided to parents was mixed. While some mentioned that the medical information supplied by surgeons was extensive, others struggled to access information from their health providers and relied on their own research skills or personal support networks. In addition to GPs, pediatricians and craniofacial specialists, some parents contacted health workers who were their friends and colleagues, while others reached out through family and friends to anyone who knew of someone whose child had been diagnosed with craniosynostosis. Parents also accessed numerous hospital and support group websites (both Australian and international) in their internet searches and, when possible, read scientific journal articles in order to find the information they could not obtain through their health providers.

The underlying message from parents was that it was difficult to source trusted, reliable information. Many also commented that this responsibility had been unfairly left to them. Although information about their child's diagnosis was generally available, they wondered whether the source was credible or content was based on subjective opinions, rather than being evidence-based, thereby impacting the information's usefulness. Their concern was that they therefore may not be accessing the most useful or credible information. The parents we spoke with all wanted to help minimize the distress that families encounter and were keen for trustworthy and easily accessible information sources to be developed.

Parents also requested that information be provided not just verbally, but also in a form that could be viewed and processed later. One format that could greatly assist families is an online, publicly available, central repository of information resources. This information hub could provide critical information such as the different types of craniosynostoses and their treatment in Australia. That is, nonsyndromic craniosynostosis (ie, metopic, sagittal, coronal and lambdoid synostosis) and syndromic conditions (eg, Crouzon, Apert, Muencke syndromes). Importantly, specific information should be provided for all types of craniosynostosis, even where frequency is low, as information is particularly scarce on less common conditions. Given that policies and processes differ across state health services in Australia, it is likely that some information and resources will need to be tailored to ensure relevancy and appropriateness across states/regions. Potentially, this could be achieved by linking to information provided on individual hospital websites.

Parents in this study identified a need to access both generic and specific health information to reduce uncertainty about what the future might hold for their family. This information included: (1) a “roadmap” or outline of the overall process; (2) directions to credible, relevant websites; (3) explanations of what specialist appointments are required and why; (4) available treatments for different types of craniosynostosis, and in which states of Australia; (5) what to expect in terms of their child's developmental stages and whether parental management of their child needs to adapt (eg, when they start to walk, or begin daycare or school); (6) how to prepare for a hospital stay (including what items to bring, available facilities, and visitor accommodation); (7) suggestions for constructive strategies and activities for parents on the day of surgery (eg, taking some “time out” from the hospital environment, physical exercise, etc.), and (8) positive stories with photos of happy, healthy children post-surgery, to help reinforce children's successes and achievements.

There are many ways this information could be provided. In the short-term, local clinics and hospitals might consider developing written fact sheets, pamphlets or brochures. Short videos by craniofacial surgeons explaining each condition and what surgery operations are available for each might also be considered. Development of a children's story book tailored to Australia that can be used to educate and inform other children about craniosynostosis, would also supplement the single US-based resource currently available which was written by parents of children with craniosynostosis.^17^ Digital based resources may also offer the timely access to health information that parents seek. Examples include a smartphone application to track progress and inform families about what to expect in upcoming stages, or workbooks that provide information about “the process” created. Given the long surgery times involved for some surgical techniques, electronic updates during surgery would allow parents to monitor their child's progress in real time. Templates could be provided that enable parents to create a journal that tracks their child's journey, with spaces for key dates, descriptions of treatment and photos, that can be used in later years to explain their child's story to them.

Although some of these suggestions would require considerable time and expenditure (eg, app development), others could be easily instituted (eg, fact sheets). Given parents’ preferences and needs may change over time, flexible communication options should be available in order for parents to select the supports that best suit them, including engaging or disengaging with support options as needed. Related to this is the fact that, currently, much of the information that families affected by craniosynostosis access, is sourced from a parent-run online Australian Facebook group. Sourcing health-related information online and in Facebook support groups is becoming increasingly common and, potentially, health providers should seek to provide input and assistance to these forums when possible^18,19^

Reflexive thematic analysis resulted in a range of rich data about support needs from parents connected to a national craniofacial organization. Nonetheless, recruiting via a number of organizations, or via hospitals directly, would likely have resulted in a different cohort (eg, those less informed who may be unaware of this particular craniofacial support option). Future work could address this limitation by adopting alternative recruitment strategies—such as by focussing on recruiting fathers and other caregivers in order to learn how their needs may differ, in addition to including more families impacted by craniofacial conditions other than craniosynostosis.

Overall, the parents we spoke with described being provided with little information about their child's condition, and the challenge of their subsequent search to learn and understand both craniosynostosis and its treatment process. Families were anxious, distressed and often fearful about their child's future. They reached out to peers who provided needed reassurance and who often provided highly personal information about their own family's journey. While some medical elements in Australia focus on parental well-being as an important aspect of the process, it was clear others did not. Creation of credible, reliable, customized, evidence-based information resources for families in Australia is critical.
